# Two Cases of Breast Cancer With Gastric Metastasis

**DOI:** 10.7759/cureus.43434

**Published:** 2023-08-13

**Authors:** Akane Ito, Masaki Nakatsubo, Ryusei Yoshino, Nana Yoshida, Masahiro Kitada

**Affiliations:** 1 Surgery, Asahikawa Medical University, Asahikawa, JPN; 2 Thoracic Surgery and Breast Surgery, Asahikawa Medical University Hospital, Asahikawa-shi, JPN; 3 Thoracic and Breast Surgery, Asahikawa Medical University, Asahikawa, JPN; 4 Thoracic Surgery and Breast Surgery, Asahikawa Medical University Hospital, Asahikwa-shi, JPN

**Keywords:** immunostaining, invasive lobular carcinoma, metastatic gastric cancer, metastasis of breast cancer, gastric metastasis of breast cancer

## Abstract

Gastric metastases from breast cancer are difficult to distinguish from primary gastric cancer. We report two cases of gastric metastasis of breast cancer with a review of the literature. In the first case, a 77-year-old woman was diagnosed with adenocarcinoma after upper gastrointestinal endoscopy, which revealed an erosive lesion in the gastric corpus. She was treated with an aromatase inhibitor and a CDK4/6 inhibitor, but five years later, she developed multiple bone metastases and gastric lesions, and she is currently receiving weekly paclitaxel (PTX) and bev. In the second case, a 63-year-old woman underwent total mastectomy and axillary lymphadenectomy [invasive lobular carcinoma (ILC)]. Eleven years after the surgery, the patient complained of pharyngeal tightness, and upper gastrointestinal endoscopy revealed a type 4 gastric tumor in the gastric body and posterior wall. In conclusion, when a patient with ILC or advanced breast cancer presents with gastric symptoms and anemia, it is important to examine and treat the patient based on the possibility of gastric metastasis.

## Introduction

Among the distant metastases of breast cancer, gastrointestinal metastases are rare, ranging from 0.3% to 8.9% of cases [1,2], with 28% of these being gastric metastases [3,4]. The diagnostic accuracy of gastric biopsy for metastases is low, and the histology is often similar to that of primary gastric cancer, making it difficult to distinguish between benign disease and differentiation, resulting in a low positive diagnosis rate [1]. Therefore, the diagnosis of gastric metastasis is often difficult. Herein, we report one case of breast cancer discovered after gastric cancer and one case in which the diagnosis of gastric metastasis changed after treatment for primary gastric cancer. This information was presented at the 20th Hokkaido Regional Meeting of the Japanese Breast Cancer Association on September 10, 2022.

## Case presentation

**Figure 1:**
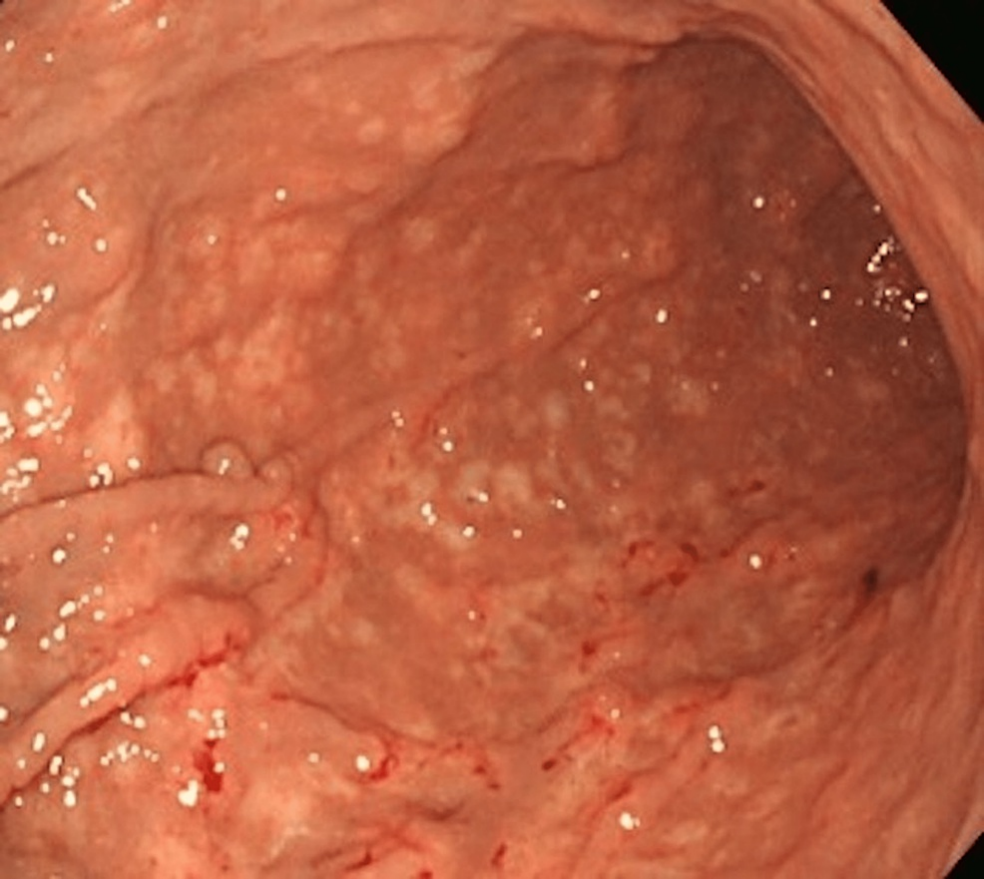
Upper endoscopy showed numerous erosions and thickened folds with some hemorrhage in the gastric body.

In case one, a 77-year-old female patient was treated for fatty liver and hepatitis B by a local physician. A routine blood draw revealed thrombocytopenia (platelet count 31.0 × 104/μL → 10.0 × 104/μL). A computed tomography (CT) scan of the patient’s entire body revealed a right breast mass (30 mm). We diagnosed the patient with invasive lobular carcinoma (ILC; positive for estrogen receptor (ER)/progesterone receptor (PgR), and negative for human epidermal growth factor receptor 2 (HER2). Upper endoscopy revealed numerous erosions and thickened folds, primarily in the gastric body. Some lesions were hemorrhagic. Although there was no evidence of dilatation, Borrmann type IV was suspected based on gross classification (Figure 1).

A biopsy of the gastric body revealed metastatic adenocarcinoma and small, atypical cells with reduced cell-cell connectivity. Tumor immunohistochemistry and morphology revealed that the patient was positive for both GATA-3 and GCDFP-15; therefore, breast cancer metastasis was suspected. The patient was resistant to an aromatase inhibitor (AI) and subsequently switched to fulvestrant; however, tumor marker elevation we observed, and the patient was referred to our department for a second opinion. As the patient refused to undergo total mastectomy, AI and palbociclib were initiated. Five years later, multiple bone metastases to the thoracolumbar spine (i.e., progressive disease; PD) and grade 3 anorexia made it difficult for the patient to continue palbociclib treatment. She has remained progression-free (i.e., stable disease).

**Figure 2:**
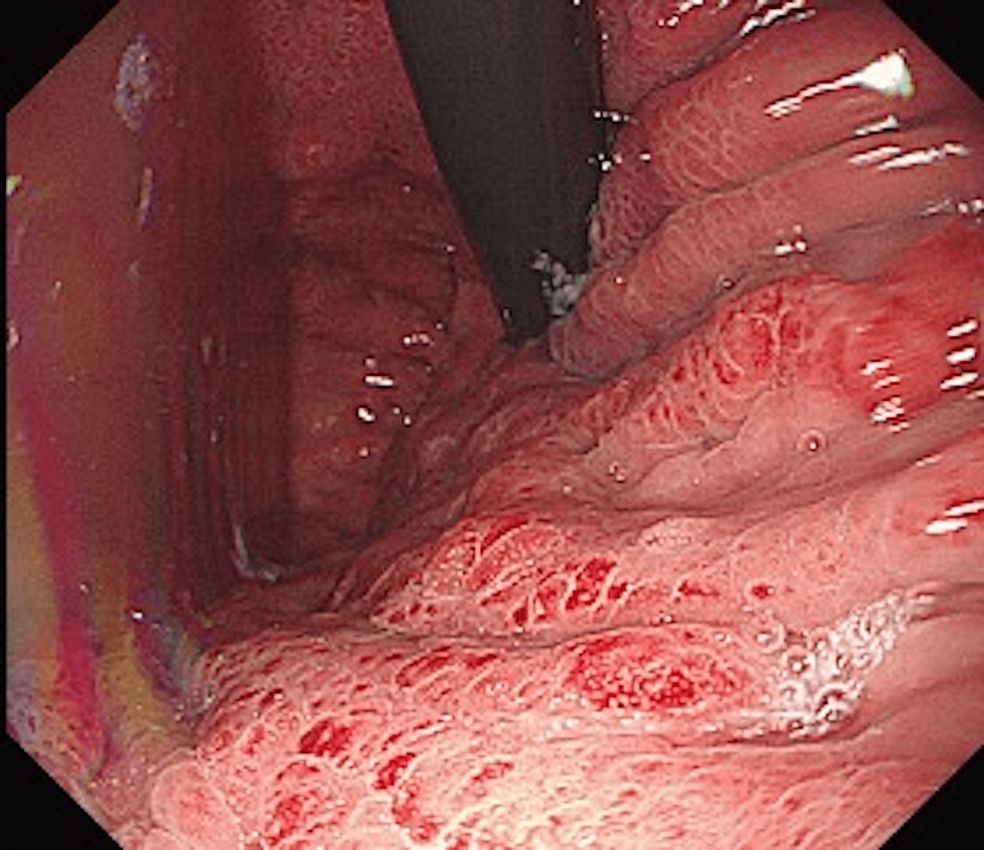
Upper endoscopy showed gastric wall thickened circumferentially, with thickened mucosa in the body of the stomach.

**Figure 3:**
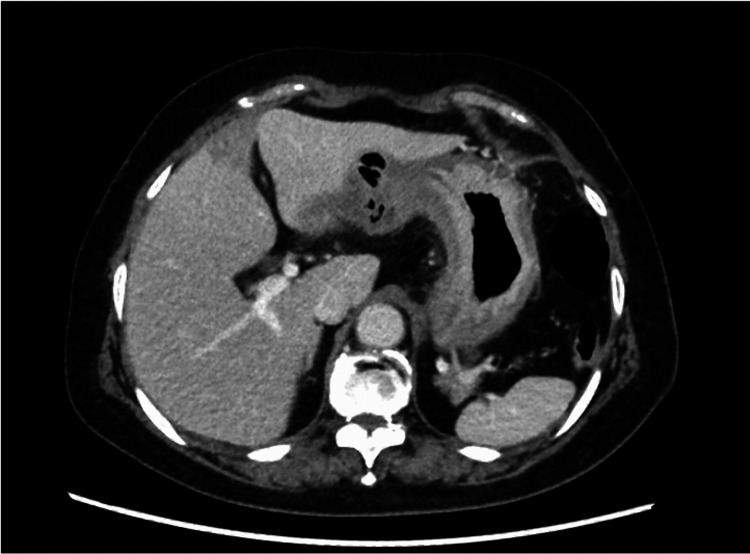
CT scan of the abdomen showed the stomach wall was thickened.

In case two, a 63-year-old female patient underwent breast-conserving surgery and sentinel node biopsy for left breast cancer. She was diagnosed with ILC (positive for ER/PgR, negative for HER2) and completed postoperative drug therapy with AI (five years), trastuzumab (one year), and radiation therapy. Ten years after completing treatment, the patient developed a pharyngeal obstruction. Upper gastrointestinal endoscopy revealed thickened folds and salmon roe-like mucosa with easy bleeding in the gastric body (Figure 2). There was no mass formation or ulceration, and Borrmann type IV was suspected based on the gross classification. A CT scan of her abdomen revealed a thickened gastric wall (Figure 3). 

**Figure 4:**
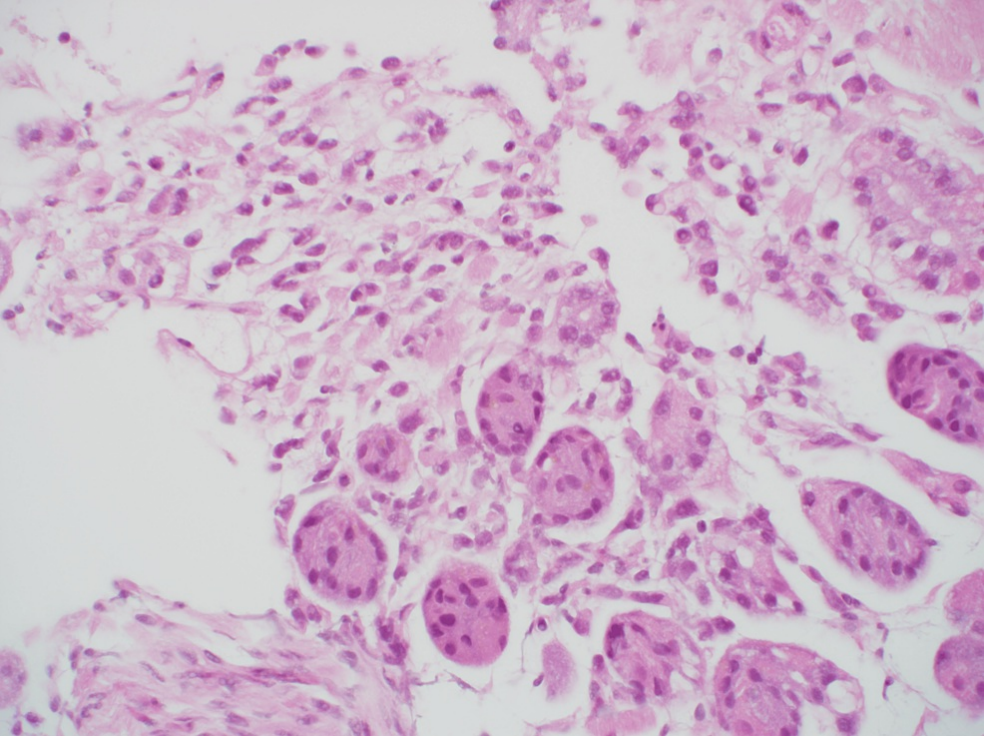
HE staining showed plasmacytoid cells infiltrating the mucosal intrinsic layer (×400). HE - hematoxylin and eosin

**Figure 5:**
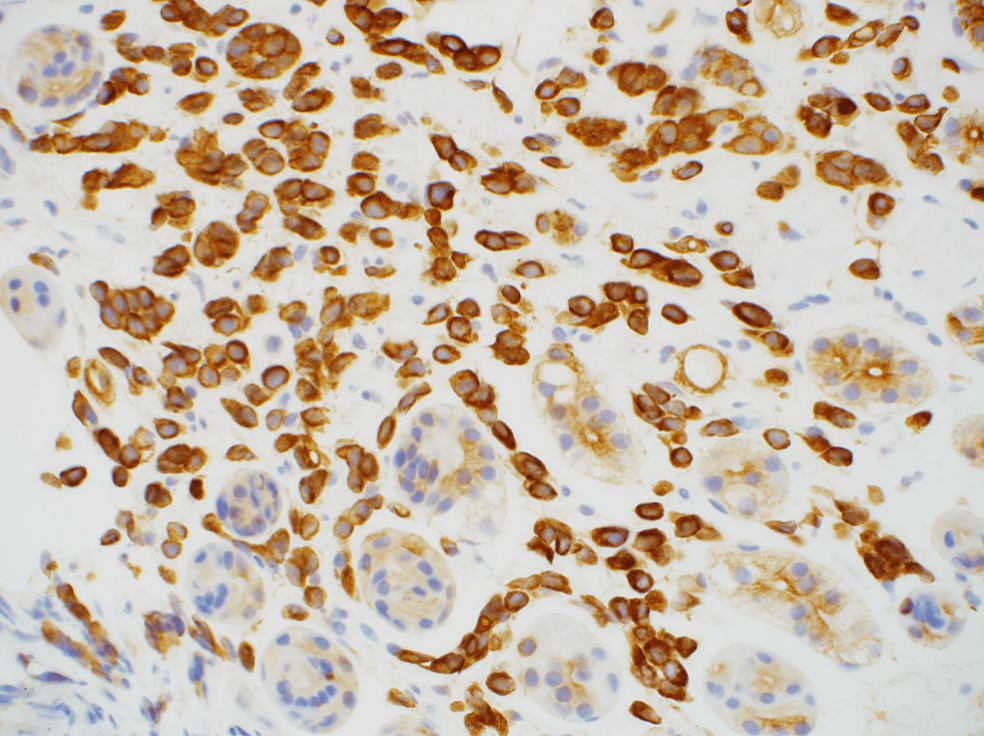
Immunohistochemistry was strongly positive for AE1/AE3 (×400).

A biopsy was performed, and hematoxylin and eosin (H&E) staining showed plasmacytoid cells infiltrating the mucosal intrinsic layer (Figure 4). Immunostaining was strongly positive for AE1/AE3 (Figure 5), and gastric cancer was diagnosed. There was no indication of gastric metastasis from the breast cancer, and ER, PgR, GATA-3, and E-cadherin levels were not evaluated. 

**Figure 6:**
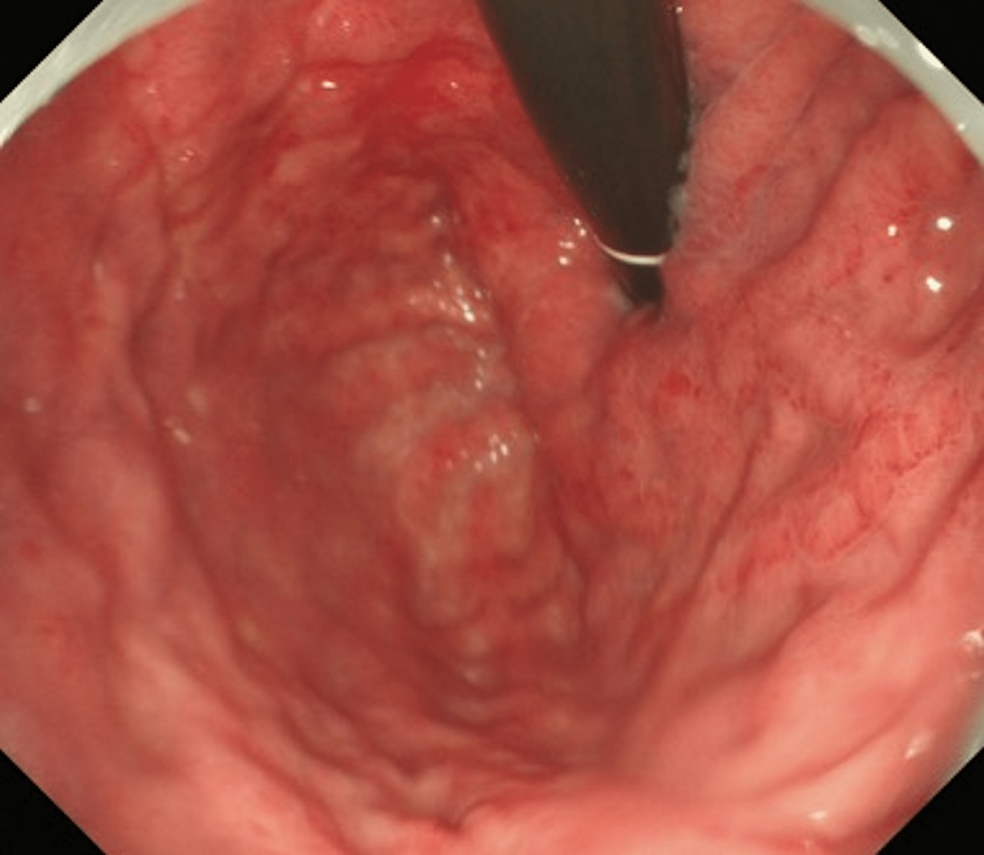
The thickened folds were enlarged circumferentially and also a puckered change in the posterior wall of the stomach. By air supply, there was some resistance in the area beyond the EGJ.

The patient was diagnosed with Borrmann type IV gastric cancer by gross classification and primary gastric cancer by biopsy (stage IIB). The patient was scheduled for laparoscopic total gastrectomy for surgical resection, but it was canceled due to intraperitoneal disseminated lesions. capecitabine + oxaliplatin (XELOX) therapy was administered, but the thickening of the gastric wall folds worsened (Figure 6). 

**Figure 7:**
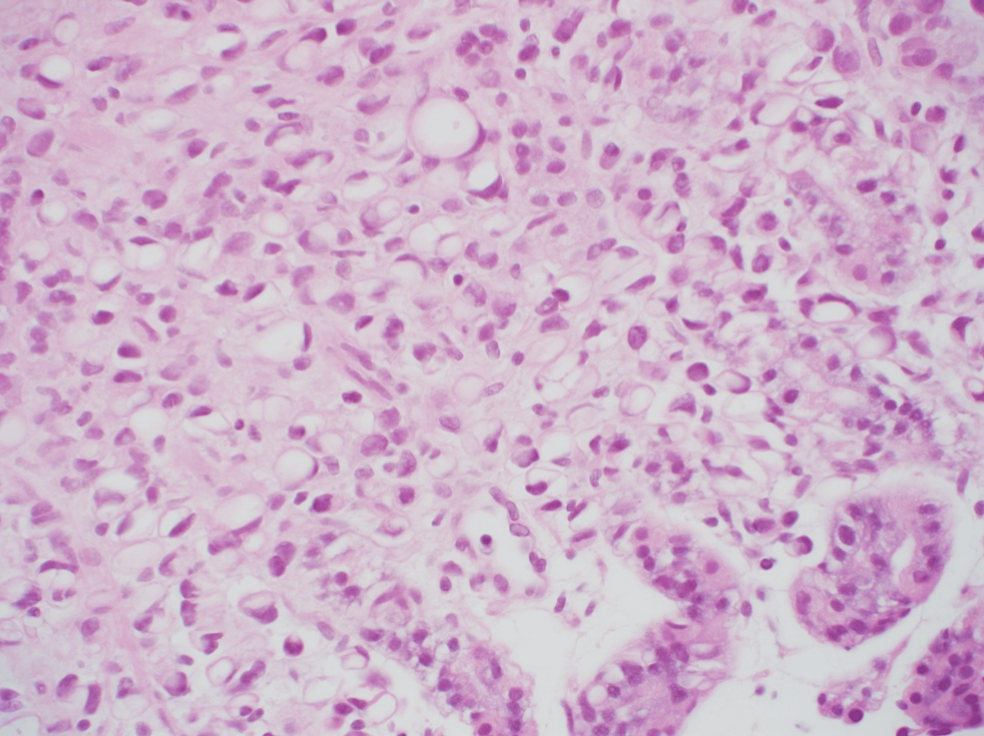
HE staining showed cancer cells with similar dysplastic cells and mucosa as seen in the breast cancer specimen (×400). HE; Hematoxylin and eosin

**Figure 8:**
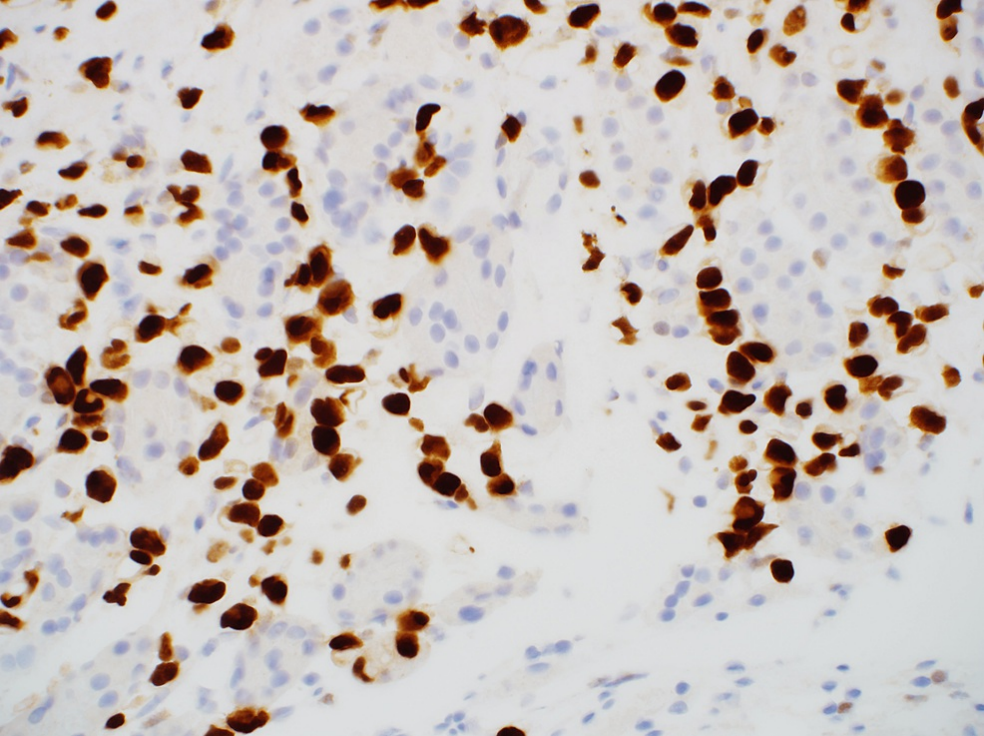
Immunohistochemistry showed GATA-3 positivity (×400).

**Figure 9:**
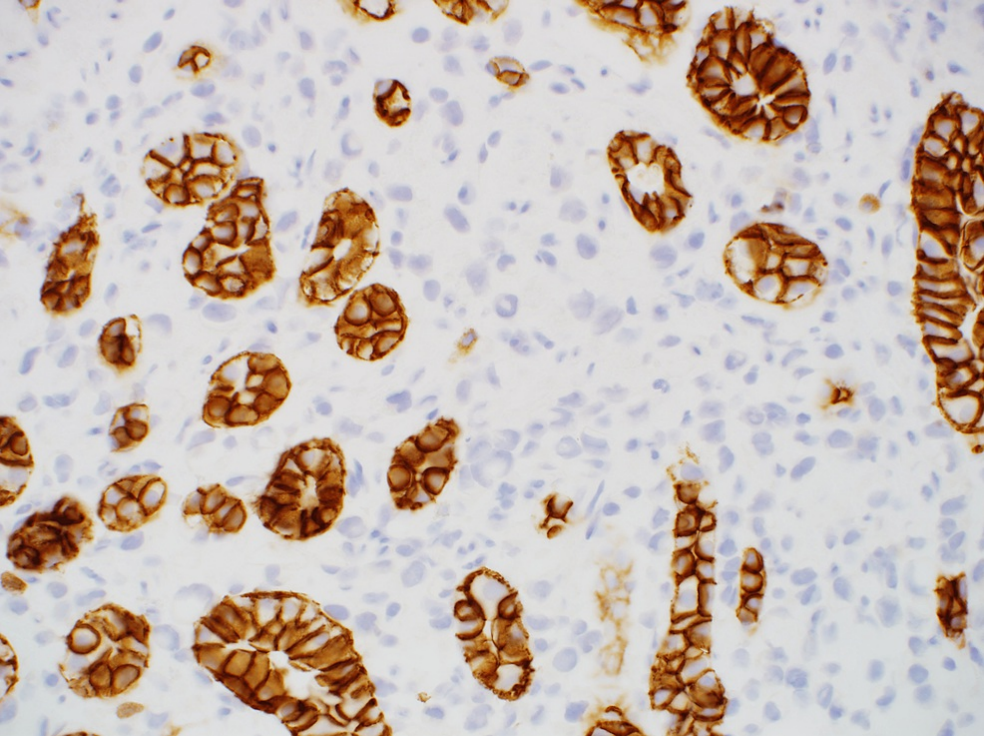
Immunohistochemistry showed E-cadherin negativity (×400).

H&E staining showed cancer cells with the same dysplastic cells and mucosa as seen in the breast cancer specimen (Figure 7). Immunostaining was positive for GATA-3 (Figure 8) and negative for E-cadherin (Figure 9), confirming the diagnosis of gastric metastasis of breast cancer. After starting fulvestrant and palbociclib, the gastric lesion was reduced (i.e., partial response), and the patient survived.

## Discussion

The lungs, bones, liver, and brain are the most common sites of distant metastasis in breast cancer. The incidence of gastric metastasis in autopsy cases of breast cancer is 7.4%-18% [1], and that in surviving breast cancer patients is 0.3%-6% [1,2], making it a rare disease. Breast cancer is the most common primary site of gastric metastasis (28%), followed by lung cancer (24%), esophageal cancer (19%), renal cell carcinoma (8%), malignant melanoma (7%), and head and neck cancer (1%-6%) [3,4]. The median survival time after diagnosis of gastric metastasis is 10 months [1], with a three-year survival rate of 79.1% [5]. The prognosis is relatively good compared to that of stage IV patients with primary gastric cancer. However, as the progression of breast cancer diagnosis to the onset of gastric metastasis typically takes five to nine years [5,6], a good prognosis can be expected if gastric metastasis, not just primary gastric cancer, is listed as a differential diagnosis in postoperative breast cancer cases, and appropriate treatment choices are made.

Clinical findings of gastric metastasis include subjective symptoms of abdominal pain, abdominal distention, bleeding, obstruction, nausea and vomiting, dysphagia, weight loss, and anemia [7]. Endoscopic findings of gastric metastasis are similar to those of primary gastric cancer, with three patterns: localized (ulcer or polyp), diffuse, and external compression, with the last often resembling diffuse inflammatory lesions with thickened folds [1]. We believe that this similarity is due to the infiltration of gastric metastases in the submucosal layer. Therefore, approximately 30% of gastric metastasis cases may be diagnosed as negative simply because the biopsy forceps do not reach the lesion [1,7].

Histopathologically, ILC accounts for 64% of gastric metastasis cases and is more common than invasive ductal carcinoma (32%) [6,8]. In addition, ILC often shows mucus-producing carcinoma cells, and its morphology is similar to that of gastric signet ring cell carcinoma, making it difficult to differentiate between the two by morphological evaluation alone in many cases [1]. Therefore, a comprehensive diagnosis that also considers immunostaining is necessary. ER and PgR, which are used as prognostic indicators for breast cancer, can be positive in 32% and 12% of gastric cancer patients, respectively, and are not suitable for differentiating between primary gastric cancer and gastric metastasis of breast cancer [9]. HER2, which is also routinely tested in primary gastric cancer, is also not useful because the HER2 type is low among lobular carcinomas (5.9% of cases) [10], and the intratumor heterogeneity of HER2 expression is higher in primary gastric cancer than in breast cancer [11]. However, case 2 was HER2-negative for metastases; morphologically similar atypical cells were observed in specimens of both cases, strongly suggesting gastric metastasis. We considered the following three hypotheses for this biomarker change: first, HER2-positive cells in the primary tumor responded to treatment, and hormone-dependent cells remained; second, HER2 heterogeneity in metastatic gastric cancer also appeared to change the biomarker; and third, XELOX therapy added some modifications to the biomarker. 

GCDFP-15 has been shown to discriminate malignant lesions of breast-cancer origin with a sensitivity of 55%-76% and a specificity of 95%-100% [12]. GATA-3 has been shown to be expressed only in breast cancer and urothelial carcinoma [13]. E-cadherin, on the other hand, has been shown to be negative in breast cancer gastric metastases [9], and CK20 has also been shown to be negative in all breast cancers [13]. As mammaglobin reportedly has a sensitivity of 55% for breast cancer but is positive in 8.1% of non-breast cancers, its utility is not certain [14]. Based on the above discussion, when breast cancer gastric metastasis is suspected, the diagnosis can be confirmed when GATA-3 and GCDFP-15 are positive, and E-cadherin and CK20 are negative. In the present case, both patients were positive for GATA-3 and negative for E-cadherin and were diagnosed with gastric metastasis from breast cancer.

Treatment options for gastric metastasis include drug therapy (e.g., systemic chemotherapy and hormonal therapy) or surgery based on metastases biomarkers. The cases in which surgery is performed include those with obstruction, perforation, bleeding, or dysphagia. At the time of diagnosis of gastric metastasis, bone metastasis (65%), peritoneal dissemination (57%), and liver metastasis (5%) are commonly already present [6], and drug therapy is the main treatment strategy [15]. Therefore, surgery is considered unnecessary in cases of gastric metastasis without bleeding or obstruction.

## Conclusions

Due to the long time to onset and low positive diagnostic rate of gastric metastasis from breast cancer, it is possible that patients are misdiagnosed with and treated for primary gastric cancer, as in case two in this report. As the prognosis for drug therapy is relatively good, it is important to differentiate gastric metastases from gastric lesions in patients with postoperative breast cancer.
